# Supplementary material for: Detection of Hepatovirus A (HAV) in wastewater indicates widespread national distribution and association with socioeconomic indicators of vulnerability
Source: mSphere. 2024 Oct 30;9(11):e00645-24. doi: 10.1128/msphere.00645-24 (PMC11580403; doi:10.1128/msphere.00645-24)
Supplement: Supplemental Material — Supplemental tables, figures, and text. [file msphere.00645-24-s0001.pdf]

Supporting information for

Detection of *Hepatovirus A* in wastewater indicates widespread national dispersion and association with socioeconomic indicators of vulnerability

Alessandro Zulli[1], Elana M. G. Chan[1], Alexandria B. Boehm[1*]

1. Department of Civil and Environmental Engineering, Stanford University, 473 Via Ortega, Stanford, California, 94305

* Corresponding author: aboehm@stanford.edu

Equation for population weighted HAV concentrations

HAV_pop_w = $\left(\sum_{i=1}^{X} pop_i * HAV_{mmwr}\right) / \left(\sum_{i=1}^{X} pop_i\right)$ (Eq 1)

Where HAV_pop_w is the state-aggregated population weighted average of HAV concentrations. Pop_i is the population served by WWTP i of X total in the state, and HAV_{mmwr} is the weekly mean of HAV gene copies per gram as grouped by Morbidity and Mortality Weekly Report (MMWR) weeks.

Multiplexing interference assessment. We sought to confirm that the multiplexing seven assays did not interfere with HAV target quantification. We tested whether the quantification of HAV in the presence of and absence of similar concentrations of the six other targets, including those for which results are not reported in this study (influenza A and B virus, the N gene of SARS-CoV-2, respiratory syncytial virus, norovirus GII, and rotavirus), was substantially different. To do so, we first quantified three decimal dilutions of a HAV nucleic acid in the absence of any other targets using the (RT)-PCR chemistry that included primers and probes for all eight targets. Then, we quantified the same decimal dilutions of the single target in the presence of 10-100 copies per reaction of the seven other targets. Each dilution was run in a single well and no template, negative controls were included on each PCR plate. Assays were run and thresholded as described in the main paper. Results were expressed as copies per reaction and the standard deviation, as output by the instrument, were included. Results are provided in Figure S1 which suggested no interference.

Additional details related to EMMI guidelines. Two-hundred six (206) wastewater samples from the study were selected at random for this analysis; this represents ~1% of the samples processed in the study. The randomly selected samples were from the samples for which 10 replicate wells were run for HAV as these raw data were relatively more easily accessible. The average (standard deviation) number of partitions (droplets) for the across 10 replicate wells was 175,792 (30,304) for the reaction for HAV. The volume of the partitions, as reported by the machine vendor is 0.00085 µL. The average (standard deviation) of copies per partition for the HAV target is 4.92×10^{-5} (1.58×10^{-4}). An example fluorescent plot from the QX600 (6 color reader) is included in the Stanford Digital Repository with the deposited wastewater data (https://purl.stanford.edu/qf850cv6453).

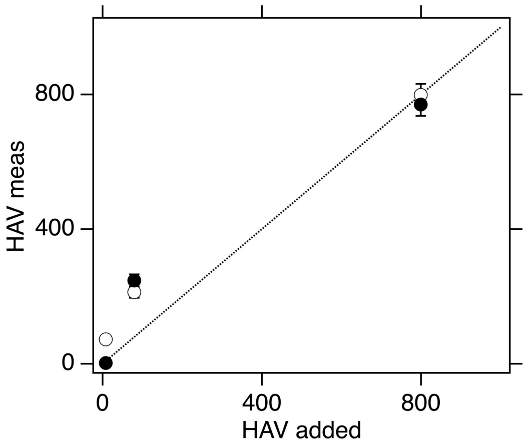

Figure S1. Multiplex assay performance. Concentrations of the HAV target in units of copies per reaction input (“HAV added”) and measured (“HAV meas”) is provided. The white symbols are for reactions without the 6 background nucleic-acid targets and black symbols include high concentrations of the 6 other nucleic-acid targets. Error bars are standard deviations, if error bars cannot be seen, then they are smaller than the symbol. The line represents the 1:1 line. NTC wells returned 0 copies of target. The markers near 0 are approximately 10 copies added.

Study Description	Environmental Sampling	Sample Treatment	Sample Reduction	Nucleic-acid Extraction	Reverse Transcription	PCR Amplification	Analysis
Study name: HAV nation<br>Date: July 2024<br>Completed by: A. Boehm	Notes: Decribed in methods section	Notes: No sample treatment performed	Notes: Centrifugation was used, as described in the methods	Notes: Methods provided in paper.	Notes: Performed one-step RT-PCR	Notes: droplet digital PCR used	Notes: Provided in methods

Control Checklist	Environmental Sampling	Sample Treatment	Sample Reduction	Nucleic-acid Extraction	Reverse Transcription	PCR Amplification	
Step performed	☑	☐	☑	☑	☑	☑	
Step has control info	☐	☐	☐	☑	☑	☑	Negative controls
# of control replicates	0	na	0	2	2	2	
Control result reported	☐	☐	☐	☑	☑	☑	
Method for handling failed controls described	☐	☐	☑	☑	☑	☑	
Step has control info	☑	☐	☑	☐	☐	☐	Positive controls
Control identity described	☑	☐	☑	☑	☑	☑	
Control quantification method described	☑	☐	☑	☑	☑	☑	
# control replicates	internal control	na	6 to 10	6 to 10	6 to 10	6 to 10	
Control result reported	☑	☐	☑	☑	☑	☑	
Method for handling failed controls described	☑	☐	☑	☑	☑	☑	

Process checklist

Environmental Sampling	
Sample procedure	☑
Number of samples	☑
Sample amount, mean, range	☑
Sampling locations, dates, times	☑
Sample storage conditions	☑
**Sample Treatment**	
Treatment procedure	☐
Reagents	☐
**Sample Reduction**	
Reduction procedure	☑
Reagents	☑
Concentration factor	☑

Nucleic-acid Extraction	
Extraction procedure	☑
Volume or mass extracted, volume or mass obtained	☑
Extract storage conditions	☑
**Reverse Transcription**	
One- or two-step	☑
cDNA storage conditions (if 2 step)	☐
Reaction temperatures and times	☑
Reaction reagents and concentrations	☑
Priming method	☑
Reaction volume, added template amount	☑
RT efficiency assessment procedure (if 2-step)	☐
RT control description (if two-step)	☐
RT efficiency reported (if 2-step)	☐

qPCR or dPCR	
Target gene name, amplicon length	☑
Thermocycling temp and times	☑
Master mix composition: vendors, concentrations	☑
Additives: vendors, composition	☑
Template amount added, pre-treatment (if any)	☑
Primers: sequences, concentrations, vendors, references	☑
Amplicon confirmation method (probe, melt curve details, etc)	☑
Probe sequence, concentration, vendor, reference	☑
Instrumentation	☑
Inhibition assessment procedure	☑
Inhibition control description (if used)	☐
Number of samples tested and found inhibited	☐
Equivalent volume of sample analyzed	☑

Analysis- dPCR	
Threshold settings	☑
Technical replicates, number, well merging	☑
Partitions measured, number, mean, variance	☑
Partition volume	☑
Target copies per partition, mean, variance	☑
Program used for dPCR analysis	☑
Explanation of control results, example plots	☑
**Analysis- qPCR**	
Technical replicates, number, calculations	☐
Calibration standards, description, source	☐
Method of quantifying standards	☐
Calibration curve slope	☐
Calibration curve R2	☐
Lowest standard measured or 95% LOD	☐
Cq value determination methods	☐

Note to users: This checklist is provided as guidance for best practices for reporting, but is not meant to be prescriptive. Not all items in the check list will apply to all studies. Please see Borchardt et al. The Environmental

Version 3.0

This version maintained by Borchardt, Boehm, Salit, Noble, Wiggington, Spencer

Date: 18 October 2023

Figure S2. EMMI guidelines[1] checklist.

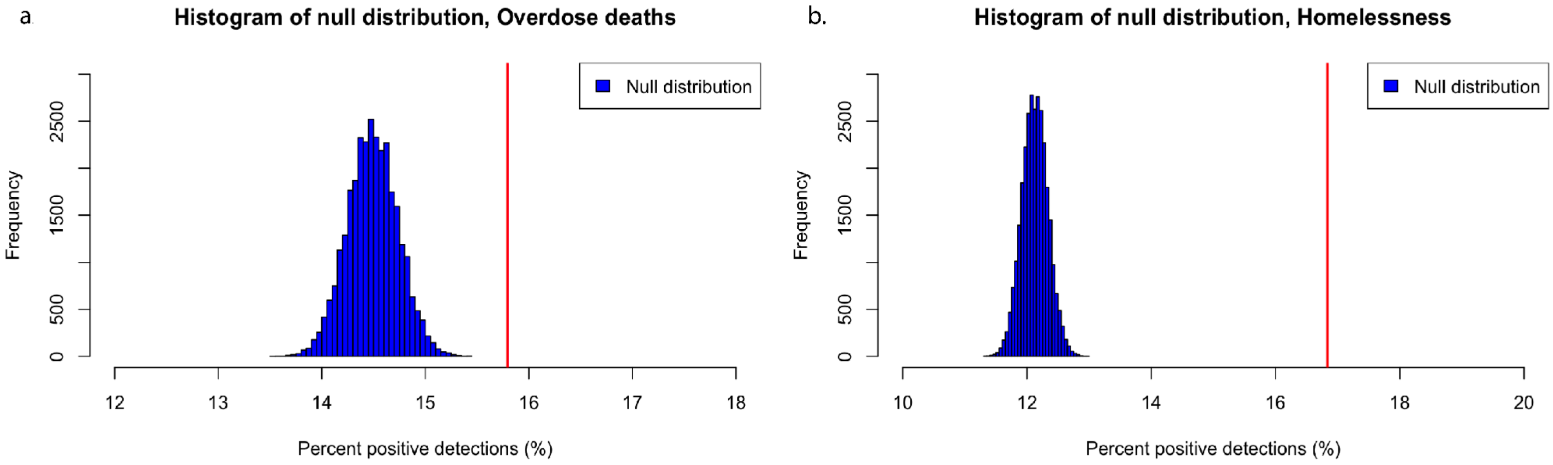

Figure S3. Null distributions compared to test statistics. Figure S3A shows the null distribution in blue bars for percent positive detections of HAV. The red line represents our test statistic. In both analyses, the test statistic is far outside the null distribution.

Table S1. Plant characteristics including sample type and population served. Plants marked with an asterisk (*) are plants where 10 replicate wells were used for digital droplet PCR, the remaining plants had 6 wells used to measure HAV, and 2 for PMMoV and BCoV. The site name, location (town, and state), state where plant is located, and whether the sample originally supplied to us was a 24 hour composite sample of liquid influent ("liquid") or a sample from the primary clarifier ("solids").

Site Name	Location	State	Sample Type	Population served
Valley Creek Water Reclamation Facility	Bessemer, AL	Alabama	Liquids	225,000
Cahaba River Water Reclamation Facility	Cahaba River, Birmingham, AL	Alabama	Liquids	95,000
Five Mile Creek Water Reclamation Facility	Fultondale, AL	Alabama	Liquids	77,000
Turkey Creek Water Reclamation Facility	Pinson, AL	Alabama	Liquids	30,000
Village Creek Water Reclamation Facility	Village Creek, Birmingham, AL	Alabama	Liquids	200,000
John M. Asplund Water Pollution Control Facility	Anchorage, AK	Alaska	Liquids	220,000
City of Harrison Wastewater Treatment Plant	Harrison, AR	Arkansas	Liquids	15,000
Coastal Treatment Plant	Coastal, Laguna Niguel, CA	California	Liquids	48,000
Central Contra Costa Sanitary District	Contra Costa County, CA	California	Solids	484,800
City of Davis Wastewater Treatment Plant	Davis, CA	California	Solids	68,000
Esparto Wastewater Treatment Facility	Esparto, CA	California	Liquids	4,006
Fairfield-Suisun Sewer District	Fairfield, CA	California	Solids	155,000
[Fremont Basin] - Raymond A. Boege Alvarado WWTP	Fremont, CA	California	Liquids	229,476
South County Regional Wastewater Authority *	Gilroy, CA	California	Solids	110,338
Sewer Authority Mid-Coastside	Half Moon Bay, CA	California	Liquids	28,000

City of Hollister Domestic Water Recycling Facility	Hollister, CA	California	Liquids	42,000
Valley Sanitary District	Indio, CA	California	Solids	91,765
JB Latham Treatment Plant	JB Latham, Laguna Niguel, CA	California	Liquids	120,000
Lancaster Water Reclamation Plant	Lancaster, CA	California	Liquids	200,000
Las Gallinas Valley Sanitary District	Las Gallinas, San Rafael, CA	California	Liquids	30,000
Lompoc Regional Wastewater Reclamation Plant	Lompoc, CA	California	Liquids	69,290
Joint Water Pollution Control Plant	Los Angeles County, CA	California	Liquids	3,500,000
Hyperion Water Reclamation Plant (HWRP)	Los Angeles, CA	California	Liquids	4,000,000
Los Banos Wastewater Treatment Plant	Los Banos, CA	California	Liquids	42,000
City of Madera, Wastewater Treatment Plant	Madera, CA	California	Solids	67,944
Mammoth Community Water District	Mammoth, CA	California	Liquids	35,000
Monterey One Water - Regional Treatment Plant	Marina, CA	California	Liquids	262,000
Merced Wastewater Treatment Plant	Merced, CA	California	Solids	91,000
Sewerage Agency of Southern Marin Wastewater Treatment Plant	Mill Valley, CA	California	Solids	30,000
Modesto's Sutter Primary Treatment Facility	Modesto, CA	California	Solids	230,000
Soscol Water Recycling Facility	Napa, CA	California	Solids	83,300
[Newark Basin] - Raymond A. Boege Alvarado WWTP	Newark, CA	California	Liquids	47,229
Novato Sanitary District	Novato, CA	California	Liquids	53,000
East Bay Municipal Utility District	Oakland, CA	California	Solids	740,000
Oceanside Water Pollution Control Plant *	Oceanside, San Francisco, CA	California	Solids	250,000

Regional Water Recycling Plant No.1 (RP-1)	Ontario, CA	California	Solids	890,000
Calera Creek Water Recycling Plant	Pacifica, CA	California	Liquids	40,000
Palo Alto Regional Water Quality Control Plant *	Palo Alto, CA	California	Solids	236,000
City of Paso Robles Wastewater Treatment Plant	Paso Robles, CA	California	Solids	31,037
Ellis Creek Water Recycling Facility	Petaluma, CA	California	Liquids	65,000
Silicon Valley Clean Water *	Redwood City, CA	California	Solids	199,000
Regional Treatment Plant	Regional, Laguna Niguel, CA	California	Liquids	129,000
Riverside Water Quality Control Plant	Riverside, CA	California	Liquids	350,000
Sacramento Regional Wastewater Treatment Plant *	Sacramento, CA	California	Solids	1,480,000
E.W. Blom Point Loma Wastewater Treatment Plant	San Diego, CA	California	Liquids	2,200,000
San Jose-Santa Clara Regional Wastewater Facility *	San Jose, CA	California	Solids	1,500,000
City of San Leandro Water Pollution Control Plant	San Leandro, CA	California	Liquids	50,000
City of San Mateo & Estero M.I.D. Water Quality Control Plant	San Mateo, CA	California	Solids	150,000
Central Marin Sanitation Agency	San Rafael, CA	California	Liquids	104,250
City of Santa Cruz WTF - County Influent	Santa Cruz County, CA	California	Solids	160,000
City of Santa Cruz WTF - City Influent	Santa Cruz, CA	California	Solids	160,000
City of Santa Rosa, Laguna Treatment Plant	Santa Rosa, CA	California	Solids	230,000
Sausalito-Marin City Sanitary District	Sausalito, CA	California	Liquids	18,000
South Bay International Wastewater Treatment Plant	South San Diego, CA	California	Solids	1,600,000
Southeast Waste Pollution Control Plant *	Southeast San Francisco, CA	California	Solids	750,000

City of Sunnyvale Water Pollution Control Plant *	Sunnyvale, CA	California	Solids	153,000
Turlock Regional Water Quality Control Facility	Turlock, CA	California	Liquids	86,000
[Union City Basin] - Raymond A. Boege Alvarado WWTP	Union City, CA	California	Liquids	68,150
Vallejo Flood and Wastewater District Wastewater Treatment Plant	Vallejo, CA	California	Liquids	121,000
West County Wastewater District	West Contra Costa County, CA	California	Liquids	100,000
Central Marin Sanitation Agency - West Railroad	West Railroad, San Rafael, CA	California	Liquids	25,000
Windsor Wastewater Treatment, Reclamation, and Disposal Facility	Windsor, CA	California	Liquids	28,000
Winters - East Street Pump Station	Winters, CA	California	Liquids	7,286
Woodland Water Pollution Control Facility	Woodland, CA	California	Liquids	59,000
Parker Water and Sanitation District North Water Reclamation Facility	North, Parker, CO	Colorado	Liquids	35,000
Parker Water and Sanitation District South Water Reclamation Facility	South, Parker, CO	Colorado	Liquids	25,000
Stamford Water Pollution Control Authority (WPCA)	Stamford, CT	Connecticut	Solids	140,000
Seaford Wastewater Treatment Facility	Seaford, DE	Delaware	Solids	13,172
Altamonte Springs Regional Water Reclamation Facility	Altamonte Springs, FL	Florida	Liquids	95,000
Eastern Water Reclamation Facility	Eastern, Orange County, FL	Florida	Solids	195,299
Loxahatchee River Environmental Control District	Jupiter, FL	Florida	Liquids	90,000
MDWASD Central District WWTP	Key Biscayne, FL	Florida	Liquids	829,725
MDWASD North District WWTF	North Miami, FL	Florida	Liquids	776,150
Northeast Water Reclamation Facility	Northeast, Saint Petersburg, FL	Florida	Liquids	89,847

Northwest Water Reclamation Facility	Northwest, Orange County, FL	Florida	Solids	66,690
Northwest Water Reclamation Facility	Northwest, Saint Petersburg, FL	Florida	Liquids	94,218
MDWASD South District WWTF	South Miami, FL	Florida	Liquids	920,528
South Water Reclamation Facility	South, Orange County, FL	Florida	Solids	183,009
Hamlin Water Reclamation Facility	Southwest, Orange County, FL	Florida	Solids	50,000
Southwest Water Reclamation Facility	Southwest, Saint Petersburg, FL	Florida	Liquids	47,790
TPSmith Water Reclamation Facility	Tallahassee, FL	Florida	Liquids	212,065
Big Creek Water Reclamation Facility	Big Creek, Roswell, GA	Georgia	Liquids	189,593
Camp Creek Water Reclamation Facility	College Park, GA	Georgia	Liquids	73,821
South Columbus Water Resources Facility	Columbus, GA	Georgia	Solids	278,000
Johns Creek Environmental Campus	Johns Creek, Roswell, GA	Georgia	Liquids	84,486
Little River Water Reclamation Facility	Little River, Roswell, GA	Georgia	Liquids	12,818
RM Clayton Water Reclamation Center	RM Clayton, Atlanta, GA	Georgia	Liquids	294,660
South River Water Reclamation Center	South River, Atlanta, GA	Georgia	Liquids	105,160
Utoy Creek Water Reclamation Center	Utoy Creek, Atlanta, GA	Georgia	Liquids	70,887
Hilo Wastewater Treatment Plant	Hilo, HI	Hawaii	Liquids	16,257
Honouliuli Wastewater Treatment Plant	Honouliuli, Honolulu, HI	Hawaii	Liquids	300,000
Kailua Regional Wastewater Treatment Plant	Kailua, Honolulu, HI	Hawaii	Liquids	90,000
Sand Island Wastewater Treatment Plant	Sand Island, Honolulu, HI	Hawaii	Liquids	390,000
Wahiawa Wastewater Treatment Plant	Wahiawa, Honolulu, HI	Hawaii	Liquids	18,000

Waianae Wastewater Treatment Plant	Waianae, Honolulu, HI	Hawaii	Liquids	44,000
City of Coeur d'Alene Water Resource Recovery Facility	Coeur d'Alene, ID	Idaho	Solids	50,540
Lander Street Water Renewal Facility	Lander Street, Boise, ID	Idaho	Liquids	108,556
West Boise Water Renewal Facility	West Boise, ID	Idaho	Liquids	186,901
Glenbard Wastewater Authority	Glen Ellyn, IL	Illinois	Solids	86,000
Wheaton Sanitary District	Wheaton, IL	Illinois	Solids	63,000
Dillman Road WWTP	Bloomington, IN	Indiana	Liquids	56,090
City of Carmel WWTP	Carmel, IN	Indiana	Solids	86,000
Jeffersonville Downtown WWTP	Downtown, Jeffersonville, IN	Indiana	Liquids	25,000
North Water Reclamation Facility	North, Jeffersonville, IN	Indiana	Liquids	25,000
City of South Bend Wastewater Treatment Plant	South Bend, IN	Indiana	Liquids	130,000
City of Clinton	Clinton, IA	Iowa	Solids	29,300
Coralville Wastewater Treatment Facility	Coralville, IA	Iowa	Liquids	23,000
City of Marshalltown Water Pollution Control Plant	Marshalltown, IA	Iowa	Liquids	27,400
Muscatine STP	Muscatine, IA	Iowa	Solids	24,400
Ottumwa WPCF	Ottumwa, IA	Iowa	Liquids	25,529
Municipal Wastewater Treatment Plant No. 1 (Kaw Point)	Kaw Point, Kansas City, KS	Kansas	Solids	90,000
Lawrence Kansas River Wastewater Treatment Facility	Lawrence, KS	Kansas	Solids	80,000
Kansas City Treatment Plant #20	P20, Kansas City, KS	Kansas	Solids	35,000
Salina Wastewater Treatment Plant	Salina, KS	Kansas	Solids	47,000
Wolcott Wastewater Treatment Facility	Wolcott, Kansas City, KS	Kansas	Liquids	15,000
Morris Forman Water Quality Treatment Center	Louisville, KY	Kentucky	Solids	423,913

SWBNO East Bank Wastewater Treatment Plant	New Orleans	Louisiana	Liquids	333,400
SWBNO West Bank Wastewater Treatment Plant	New Orleans	Louisiana	Liquids	50,500
City of Bangor Wastewater Treatment Plant	Bangor, ME	Maine	Solids	40,000
Brunswick Sewer District	Brunswick, ME	Maine	Liquids	10,000
Lewiston Auburn Water Pollution Control Authority	Lewiston, ME	Maine	Liquids	60,000
Portland Water District (East End Wastewater Treatment Facility)	Portland, ME	Maine	Liquids	65,000
York Sewer District	York, ME	Maine	Liquids	10,000
Hagerstown Wastewater Treatment Plant	Hagerstown, MD	Maryland	Liquids	90,000
Marlay Taylor Water Reclamation Facility	Hollywood, MD	Maryland	Liquids	55,000
Deer Island Treatment Plant	Boston, MA	Massachu setts	Solids	2,400,000
Upper Blackstone Clean Water	Millbury, MA	Massachu setts	Liquids	250,000
City of Ann Arbor Wastewater Treatment Plant	Ann Arbor, MI	Michigan	Liquids	125,000
Jackson Wastewater Treatment Plant	Jackson, MI	Michigan	Solids	90,000
Grandville Clean Water Plant	Jenison, MI	Michigan	Solids	75,000
Mt. Pleasant WRRF	Mt. Pleasant, MI	Michigan	Liquids	21,690
Traverse City Regional Waste Water Treatment Plant	Traverse City, MI	Michigan	Liquids	30,623
City of Warren Wastewater Treatment Plant	Warren, MI	Michigan	Liquids	140,000
City of Mankato Water Resource Recovery Facility (WRRF)	Mankato, MN	Minnesot a	Solids	70,000
Red Wing Wastewater Treatment Facility	Red Wing, MN	Minnesot a	Solids	16,000

City Of Rochester MN Water Reclamation Plant	Rochester, MN	Minnesota	Solids	120,000
St. Cloud Nutrient, Energy and Water Recovery Facility	St. Cloud, MN	Minnesota	Liquids	120,000
7 C- Pascagoula Moss Point POTW (Vancleave, MS)	Jackson County, MS	Mississippi	Liquids	34,333
2C-Gautier POTW (Vancleave, MS)	Jackson County, MS	Mississippi	Liquids	19,008
Northeast Water Resource Recovery Facility	Northeast, Lincoln, NE	Nebraska	Liquids	60,000
Theresa Street Water Resource Recovery Facility	Theresa Street, Lincoln, NE	Nebraska	Liquids	240,000
Clark County Water Reclamation District (CCWRD) Flamingo Water Resource Center (FWRC)	Las Vegas, NV	Nevada	Liquids	990,000
City of Dover Wastewater Treatment Facility	Dover, NH	New Hampshire	Liquids	30,000
Hall Street Wastewater Treatment Plant	Hall Street, Concord, NH	New Hampshire	Liquids	45,000
Penacook Wastewater Treatment Facility	Penacook, Concord, NH	New Hampshire	Liquids	4,000
South Monmouth Regional Sewerage Authority	Belmar, NJ	New Jersey	Solids	52,672
Cumberland County Utilities Authority	Bridgeton, NJ	New Jersey	Liquids	50,000
The Somerset Raritan Valley Sewerage Authority	Bridgewater, NJ	New Jersey	Liquids	130,000
Passaic Valley Sewerage Commission	Newark, NJ	New Jersey	Solids	1,500,000
Township of Ocean Sewerage Authority	Oakhurst, NJ	New Jersey	Liquids	50,000
Bayshore Regional Sewerage Authority	Union Beach, NJ	New Jersey	Solids	100,000

Ithaca Area Wastewater Treatment Facility	Ithaca, NY	New York	Liquids	90,000
City of Oswego Wastewater Treatment Plant	Oswego, NY	New York	Liquids	30,000
Johnnie Mosley Regional Water Reclamation Facility	Kinston, NC	North Carolina	Liquids	25,000
City of Wilson - Hominy Creek Water Reclamation Facility	Wilson, NC	North Carolina	Solids	50,000
Archie Elledge WWTP	Winston-Salem, NC	North Carolina	Liquids	92,000
Akron Water Reclamation Facility	Akron, OH	Ohio	Solids	365,000
City of Youngstown Wastewater Treatment Plant	Youngstown, OH	Ohio	Solids	174,000
DELCORA Western Regional Treatment Plant	Chester, PA	Pennsylvania	Liquids	220,000
Capital Region Water AWTF	Harrisburg, PA	Pennsylvania	Liquids	125,000
Penn State Water Treatment Facility	Penn State University Park	Pennsylvania	Liquids	16,000
City of Yankton Wastewater Treatment Facility	Yankton, SD	South Dakota	Liquids	20,000
Moccasin Bend WWTP	Chattanooga, TN	Tennessee	Liquids	400,000
M.C. Stiles Wastewater Treatment Facility	Memphis, TN	Tennessee	Liquids	300,000
DCWT Dallas	Dallas Central, Dallas, TX	Texas	Liquids	270,000
City of Gainesville Wastewater Treatment Plant	Gainesville, TX	Texas	Liquids	17,300
City of Garland Rowlett Creek WWTP	Garland, TX	Texas	Solids	200,000
Hollywood Road WWTP	Hollywood Road, Amarillo, TX	Texas	Liquids	60,000
River Road WWTP	River Road, Amarillo, TX	Texas	Liquids	140,000
South Laredo WWTP	South, Laredo, TX	Texas	Liquids	120,000
Southside Wastewater Treatment Plant (City of Dallas)	Southside, Dallas, TX	Texas	Liquids	421,700

Duck Creek Wastewater Treatment Plant	Sunnyvale, TX	Texas	Solids	186,000
DCWT White Rock	White Rock Central, Dallas, TX	Texas	Liquids	630,000
Wichita Falls Resource Recovery Facility	Wichita Falls, TX	Texas	Solids	90,000
SJRA WWTF No.1	Woodlands SJRA WWTF No. 1, TX	Texas	Liquids	65,000
SJRA WWTF No.2	Woodlands SJRA WWTF No. 2, TX	Texas	Liquids	70,000
SJRA WWTF No.3	Woodlands SJRA WWTF No. 3, TX	Texas	Liquids	15,000
Zacate Creek WWTP	Zacate Creek, Laredo, TX	Texas	Liquids	140,000
Central Valley Water Reclamation Facility	Central Salt Lake Valley, UT	Utah	Solids	600,000
Provo City Water Reclamation Facility	Provo, UT	Utah	Solids	115,000
City of Essex Junction Wastewater Treatment Facility	Essex Junction, VT	Vermont	Solids	30,000
Montpelier Water Resource Recovery Facility	Montpelier, VT	Vermont	Solids	10,100
South Burlington-Airport Parkway WWTF	South Burlington, VT	Vermont	Liquids	16,000
Aquia Wastewater Treatment Facility	Aquia, Stafford, VA	Virginia	Solids	100,000
Town of Hillsville Wastewater Treatment Plant	Hillsville, VA	Virginia	Solids	3,000
Little Falls Run Wastewater Treatment Facility	Little Falls Run, Stafford, VA	Virginia	Solids	50,000
City of Snohomish WWTP	Snohomish, WA	Washington	Liquids	10,150
City of Wheeling, Water Pollution Control Division	Wheeling, WV	West Virginia	Liquids	100,000
Wausau Waterworks Wastewater Treatment Facility	Wausau, WI	Wisconsin	Solids	44,000

Blue Plains Advanced Wastewater Treatment Tlant	Washington DC	District of Columbia	Liquids	2,000,000

Table S2. Percentage of samples positive for HAV by plant. All samples collected during the study period were aggregated for each treatment plant. Plants are ordered by state and then alphabetically.

**Wastewater Treatment Plant**	**State**	**Percentage of samples positive for HAV (%)**	**n**
John M. Asplund Water Pollution Control Facility	AK	5.35714286	112
Mendenhall Wastewater Treatment Plant	AK	0	1
Cahaba River Water Reclamation Facility	AL	0	115
Five Mile Creek Water Reclamation Facility	AL	0.88495575	113
Turkey Creek Water Reclamation Facility	AL	3.63636364	110
Valley Creek Water Reclamation Facility	AL	0.86956522	115
Village Creek Water Reclamation Facility	AL	27.3584906	106
City of Harrison Wastewater Treatment Plant	AR	1.75438596	114
A.K. Warren Water Resource Facility	CA	60.5263158	114
CODIGA	CA	0	235
Calera Creek Water Recycling Plant	CA	0	29
Central Contra Costa Sanitary District	CA	15.8878505	107
Central Marin Sanitation	CA	0	69

Agency			
City of Davis Wastewater Treatment Plant	CA	0.91743119	109
City of Hollister Domestic Water Recycling Facility	CA	0	106
City of Madera, Wastewater Treatment Plant	CA	0	109
City of Paso Robles Wastewater Treatment Plant	CA	5.35714286	112
City of San Leandro Water Pollution Control Plant	CA	4.34782609	115
City of San Mateo & Estero M.I.D. Water Quality Control Plant	CA	10.5263158	114
City of Santa Cruz WTF - City Influent	CA	0.89285714	112
City of Santa Cruz WTF - County Influent	CA	0	112
City of Santa Rosa, Laguna Treatment Plant	CA	47.706422	109
City of Sunnyvale Water Pollution Control Plant	CA	48.4848485	264
Coastal Treatment Plant	CA	0	115
E.W. Blom Point Loma Wastewater Treatment Plant	CA	37.1681416	113
East Bay Municipal Utility District	CA	34.2105263	114
Ellis Creek Water Recycling Facility	CA	2.77777778	36
Esparto Wastewater Treatment Facility	CA	0	116
Fairfield-Suisun Sewer District	CA	20	115
Hyperion Water Reclamation Plant (HWRP)	CA	71.0526316	114

JB Latham Treatment Plant	CA	3.44827586	116
Lancaster Water Reclamation Plant	CA	0	103
Las Gallinas Valley Sanitary District	CA	12.2807018	114
Lompoc Regional Wastewater Reclamation Plant	CA	6.14035088	114
Los Banos Wastewater Treatment Plant	CA	0	106
Mammoth Community Water District	CA	0	109
Merced Wastewater Treatment Plant	CA	6.08695652	115
Modesto's Sutter Primary Treatment Facility	CA	3.6036036	111
Monterey One Water - Regional Treatment Plant	CA	14.5631068	103
Novato Sanitary District	CA	1.72413793	116
Oceanside Water Pollution Control Plant	CA	24.4186047	258
Palo Alto Regional Water Quality Control Plant	CA	33.5766423	274
Regional Treatment Plant	CA	0	115
Regional Water Recycling Plant No.1 (RP-1)	CA	26.7857143	112
Riverside Water Quality Control Plant	CA	7.82608696	115
Sacramento Regional Wastewater Treatment Plant	CA	32.1167883	274
San Jose-Santa Clara Regional Wastewater Facility	CA	52	275
Sausalito-Marin City Sanitary District	CA	1.92307692	104

Sewer Authority Mid-Coastside	CA	14.4144144	111
Sewerage Agency of Southern Marin Wastewater Treatment Plant	CA	4.34782609	115
Silicon Valley Clean Water	CA	37.6	250
Soscol Water Recycling Facility	CA	1.75438596	114
South County Regional Wastewater Authority	CA	3.27272727	275
Southeast San Francisco	CA	54.6125461	271
Turlock Regional Water Quality Control Facility	CA	4.34782609	115
Vallejo Flood and Wastewater District Wastewater Treatment Plant	CA	13.0434783	115
Valley Sanitary District	CA	16.5217391	115
West County Wastewater District	CA	1.03092784	97
Windsor Wastewater Treatment, Reclamation, and Disposal Facility	CA	0	105
Winters - East Street Pump Station	CA	0	105
Woodland Water Pollution Control Facility	CA	1.05263158	95
[Fremont Basin] - Raymond A. Boege Alvarado WWTP	CA	7.35294118	68
[Newark Basin] - Raymond A. Boege Alvarado WWTP	CA	0	75
[Union City Basin] - Raymond A. Boege Alvarado WWTP	CA	1.33333333	75
Parker Water and Sanitation District North Water Reclamation Facility	CO	1.88679245	106

Parker Water and Sanitation District South Water Reclamation Facility	CO	3.77358491	106
City of Stamford, Water Pollution Control Authority	CT	8.33333333	84
Blue Plains Advanced Wastewater Treatment Plant	DC	27.1604938	81
Seaford Wastewater Treatment Facility	DE	2.60869565	115
Altamonte Springs Regional Water Reclamation Facility	FL	15.4545455	110
Eastern Water Reclamation Facility	FL	18.9473684	95
Hamlin Water Reclamation Facility	FL	7.60869565	92
Loxahatchee River Environmental Control District	FL	21.9298246	114
MDWASD Central District WWTP	FL	57	100
MDWASD North District WWTF	FL	66.0377358	106
MDWASD South District WWTF	FL	15.5555556	45
Northeast Water Reclamation Facility	FL	0	109
Northwest Water Reclamation Facility	FL	1.96078431	204
South Water Reclamation Facility	FL	22.9166667	96
Southwest Water Reclamation Facility	FL	6.08695652	115
TPSmith Water Reclamation Facility	FL	1.78571429	112
Big Creek Water Reclamation Facility	GA	9.56521739	115

Camp Creek Water Reclamation Facility	GA	3.80952381	105
Johns Creek Environmental Campus	GA	0	114
Little River Water Reclamation Facility	GA	11.4035088	114
RM Clayton Water Reclamation Center	GA	25.5319149	94
South Columbus Water Resources Facility	GA	6.81818182	88
South River Water Reclamation Center	GA	6.38297872	94
Utoy Creek Water Reclamation Center	GA	0	94
Hilo Wastewater Treatment Plant	HI	0	53
Honouliuli Wastewater Treatment Plant	HI	0	115
Kailua Regional Wastewater Treatment Plant	HI	1.73913043	115
Sand Island Wastewater Treatment Plant	HI	3.47826087	115
Wahiawa Wastewater Treatment Plant	HI	0.88495575	113
Waianae Wastewater Treatment Plant	HI	0	115
City of Clinton	IA	0	115
City of Marshalltown Water Pollution Control Plant	IA	0.9009009	111
Coralville Wastewater Treatment Facility	IA	1.75438596	114
Muscatine STP	IA	0	100
Ottumwa WPCF	IA	0.86956522	115
City of Coeur d'Alene Water	ID	1.03092784	97

Resource Recovery Facility			
Lander Street Water Renewal Facility	ID	0.87719298	114
West Boise Water Renewal Facility	ID	3.53982301	113
Glenbard Wastewater Authority	IL	1.85185185	108
Wheaton Sanitary District	IL	5.50458716	109
City of Carmel WWTP	IN	5.2173913	115
City of South Bend Wastewater Treatment Plant	IN	12.2807018	114
Dillman Road WWTP	IN	4.03225806	124
Jeffersonville Downtown WWTP	IN	0	114
North Water Reclamation Facility	IN	0.87719298	114
Kansas City Treatment Plant #20	KS	3.2967033	91
Lawrence Kansas River Wastewater Treatment Facility	KS	85.7142857	112
Municipal Wastewater Treatment Plant No. 1 (Kaw Point)	KS	25.6880734	109
Salina Wastewater Treatment Plant	KS	48.6956522	115
Wolcott Wastewater Treatment Facility	KS	31.7307692	104
Morris Forman Water Quality Treatment Center	KY	27.0833333	96
SWBNO East Bank Wastewater Treatment Plant	LA	13.5802469	81
SWBNO West Bank Wastewater Treatment Plant	LA	23.75	80

Deer Island Treatment Plant	MA	88.5964912	114
Upper Blackstone Clean Water	MA	2.85714286	105
Hagerstown Wastewater Treatment Plant	MD	0.86956522	115
Marlay Taylor Water Reclamation Facility	MD	0	100
Brunswick Sewer District	ME	9.70873786	103
City of Bangor Wastewater Treatment Plant	ME	8.41121495	107
Lewiston Auburn Water Pollution Control Authority	ME	67.3469388	98
Portland Water District (East End Wastewater Treatment Facility)	ME	95.1807229	83
York Sewer District	ME	10	100
City of Ann Arbor Wastewater Treatment Plant	MI	18.4210526	114
City of Warren Wastewater Treatment Plant	MI	1.04166667	96
Grandville Clean Water Plant	MI	0	115
Jackson Wastewater Treatment Plant	MI	0	115
Mt. Pleasant WRRF	MI	1.85185185	108
Traverse City Regional Waste Water Treatment Plant	MI	25.2252252	111
City Of Rochester MN Water Reclamation Plant	MN	2.7027027	111
City of Mankato Water Resource Recovery Facility (WRRF)	MN	21.0526316	114
Red Wing Wastewater Treatment Facility	MN	0.87719298	114
St. Cloud Nutrient, Energy	MN	1.73913043	115

and Water Recovery Facility			
2C-Gautier POTW	MS	0	72
7 C- Pascagoula Moss Point POTW	MS	0	71
Archie Elledge WWTP	NC	16.1904762	105
City of Wilson - Hominy Creek Water Reclamation Facility	NC	0.95238095	105
Johnnie Mosley Regional Water Reclamation Facility	NC	0.95238095	105
Northeast Water Resource Recovery Facility	NE	0	115
Theresa Street Water Resource Recovery Facility	NE	0.86956522	115
City of Dover Wastewater Treatment Facility	NH	0	111
Hall Street Wastewater Treatment Plant	NH	12.1495327	107
Penacook Wastewater Treatment Facility	NH	0	105
Bayshore Regional Sewerage Authority	NJ	0.86956522	115
Cumberland County Utilities Authority	NJ	8.65384615	104
Passaic Valley Sewerage Commission	NJ	57.9545455	88
South Monmouth Regional Sewerage Authority	NJ	10.4347826	115
The Somerset Raritan Valley Sewerage Authority	NJ	0	109
Township of Ocean Sewerage Authority	NJ	0	80
Clark County Water Reclamation District (CCWRD) Flamingo Water	NV	18.1034483	116

Resource Center (FWRC)			
City of Oswego Wastewater Treatment Plant	NY	0.88495575	113
Ithaca Area Wastewater Treatment Facility	NY	7.77777778	90
Akron Water Reclamation Facility	OH	6.42201835	109
City of Youngstown Wastewater Treatment Plant	OH	0	113
Capital Region Water AWTF	PA	39.6551724	116
DELCORA Western Regional Treatment Plant	PA	53.6842105	95
University Park Water Reclamation Plant	PA	0	72
City of Yankton Wastewater Treatment Facility	SD	0.86956522	115
M.C. Stiles Wastewater Treatment Facility	TN	31.4814815	108
Moccasin Bend WWTP	TN	23.1481481	108
City of Gainesville Wastewater Treatment Plant	TX	0	107
City of Garland Rowlett Creek WWTP	TX	4.71698113	106
DCWT Dallas	TX	23.364486	107
DCWT White Rock	TX	28.7037037	108
Duck Creek Wastewater Treatment Plant	TX	23.8636364	88
Hollywood Road WWTP	TX	1.7699115	113
River Road WWTP	TX	4.23728814	118
SJRA WWTF No.1	TX	2.60869565	115
SJRA WWTF No.2	TX	1.75438596	114
SJRA WWTF No.3	TX	0	114

South Laredo WWTP	TX	13.592233	103
Southside Wastewater Treatment Plant (City of Dallas)	TX	27.7777778	90
Wichita Falls Resource Recovery Facility	TX	2.7027027	111
Zacate Creek WWTP	TX	37.8640777	103
Central Valley Water Reclamation Facility	UT	52.2123894	113
Provo City Water Reclamation Facility	UT	5.40540541	74
Aquia Wastewater Treatment Facility	VA	4.42477876	113
Little Falls Run Wastewater Treatment Facility	VA	15.9292035	113
Town of Hillsville Wastewater Treatment Plant	VA	21.4285714	112
City of Essex Junction Wastewater Treatment Facility	VT	0	114
Montpelier Water Resource Recovery Facility	VT	0.91743119	109
South Burlington-Airport Parkway WWTF	VT	0	67
City of Snohomish Wastewater Treatment Plant	WA	0	97
Wausau Waterworks Wastewater Treatment Facility	WI	5.26315789	114
City of Wheeling, Water Pollution Control Division	WV	3.73831776	107

References

(1) Borchardt, M. A.; Boehm, A. B.; Salit, M.; Spencer, S. K.; Wigginton, K. R.; Noble, R. T. The Environmental Microbiology Minimum Information (EMMI) Guidelines: qPCR and dPCR Quality and Reporting for Environmental Microbiology. *Environ. Sci. Technol.* **2021**, *55* (15), 10210–10223. https://doi.org/10.1021/acs.est.1c01767.
